# Pancreatic acinar cell fate relies on system x_C_^-^ to prevent ferroptosis during stress

**DOI:** 10.1038/s41419-023-06063-w

**Published:** 2023-08-21

**Authors:** Zhaolong Pan, Jan-Lars Van den Bossche, Eva Rodriguez-Aznar, Pauline Janssen, Olaya Lara, Gamze Ates, Ann Massie, Diedert Luc De Paep, Isabelle Houbracken, Marco Mambretti, Ilse Rooman

**Affiliations:** 1 Laboratory for Medical and Molecular Oncology, Oncology Research Center, Vrije Universiteit Brussel, Brussels, Belgium; 2 Neuro-Aging & Viro-Immunotherapy Research Group, Vrije Universiteit Brussel, Brussels, Belgium; 3 Beta Cell Bank, Universitair Ziekenhuis Brussel and Diabetes Research Center, Vrije Universiteit Brussel, Brussels, Belgium; 4 Visual and Spatial Tissue Analysis (VSTA) Core Facility, Vrije Universiteit Brussel, Brussels, Belgium

**Keywords:** Molecular biology, Pathogenesis, Diseases

## Abstract

Acinar cell dedifferentiation is one of the most notable features of acute and chronic pancreatitis. It can also be the initial step that facilitates pancreatic cancer development. In the present study, we further decipher the precise mechanisms and regulation using primary human cells and murine experimental models. Our RNAseq analysis indicates that, in both species, early acinar cell dedifferentiation is accompanied by multiple pathways related to cell survival that are highly enriched, and where *SLC7A11* (xCT) is transiently upregulated. xCT is the specific subunit of the cystine/glutamate antiporter system x_C_^-^. To decipher its role, gene silencing, pharmacological inhibition and a knock-out mouse model were used. Acinar cells with depleted or reduced xCT function show an increase in ferroptosis relating to lipid peroxidation. Lower glutathione levels and more lipid ROS accumulation could be rescued by the antioxidant N-acetylcysteine or the ferroptosis inhibitor ferrostatin-1. In caerulein-induced acute pancreatitis in mice, xCT also prevents lipid peroxidation in acinar cells. In conclusion, during stress, acinar cell fate seems to be poised for avoiding several forms of cell death. xCT specifically prevents acinar cell ferroptosis by fueling the glutathione pool and maintaining ROS balance. The data suggest that xCT offers a druggable tipping point to steer the acinar cell fate in stress conditions.

## Introduction

Acinar cell dedifferentiation and acquisition of a duct cell-like phenotype, also called acinar to ductal metaplasia (ADM), is a protective mechanism of pancreatic acinar cells, where losing their differentiated phenotype upon stress supports their survival [1, 2]. In fact, acinar cell dedifferentiation events are one of the main hallmarks of acute and chronic pancreatitis. This process is due to the high plasticity of acinar cells, and has a crucial role in pancreatic regeneration upon mild damage [1–3]. However, many studies have also demonstrated that dedifferentiated acinar cells, in contrast to fully differentiated acinar cells, are susceptible to oncogenic *KRAS* mutations, which may further give rise to pancreatic ductal adenocarcinoma (PDAC) [4–8]. Consequently, when in presence of the oncogene, acinar cell dedifferentiation is (at least partially) regarded as the initial step of PDAC development [9, 10]. Although this dynamic process is starting to be well described, the acinar cell fate under stress and the mechanism and regulation are not yet fully known. Therefore, a better understanding of how acinar cells cope with stress and can dedifferentiate could be of great help to study pancreatic tumorigenesis and develop a therapeutic intervention.

System x_C_^-^ is a cystine/glutamate antiporter which imports extracellular cystine in exchange for glutamate. The *SLC7A11* gene encoded xCT protein is its functional subunit. xCT is known to be closely involved in regulating redox homeostasis in many cells [11–13]. By transporting cystine into cells, it helps to maintain the availability of cysteine, which is necessary for the synthesis of glutathione, a potent antioxidant. Glutathione peroxidase 4 (GPX4) then utilizes glutathione as the substrate to scavenge reactive oxygen species (ROS) and thus prevents oxidative damage.

Dysregulation of cystine antiporter activity has been found to be associated with many diseases, including cancer. Interestingly, xCT expression is generally overexpressed in cancer cells, especially in pancreatic cancer [13]. This might be explained by the high metabolic rate and the need for ROS scavenging in these rapidly proliferating cells. Recent studies targeting xCT have been mainly focused on established tumors rather than tumor development, and xCT disruption was proven to cause ferroptosis in different types of cancer cells [14, 15]. Ferroptosis is a form of programmed cell death that is triggered by the accumulation of lipid peroxides in cells. It is also featured by the accumulation of iron, which leads to the production of ROS and the subsequent destabilization of cell membranes [16]. Ferroptosis is distinct from other forms of cell death (e.g., apoptosis) and can be reversed by a ferroptosis inhibitor (e.g., ferrostatin-1). Due to its antiporter function, xCT is generally known as a suppressor of ferroptosis [17, 18].

To date, apoptosis has been regarded as the main cause of cell death during the acinar cell dedifferentiation process [19], and our recent work confirmed that acinar cells during stress avoid apoptosis by induction of MECOM expression [20]. Based on an unbiased approach and considering data from primary human cells and murine cells, we now add that xCT is specifically upregulated in dedifferentiated acinar cells. We investigated the specific role of xCT in the acinar cell fate. Both in vitro and in vivo models underscore that, across the two species, xCT prevents pancreatic acinar cells from undergoing ferroptosis. By extension, we observe that the acinar cell dedifferentiation program is specifically tuned to avoid acinar cell death, a prerequisite for ADM.

## Materials and methods

### Primary cell culture

Healthy human donor pancreatic exocrine cells were obtained from the Beta Cell Bank of the JDRF Center for Beta Cell Therapy in Diabetes (Brussels, Belgium), affiliated to the Eurotransplant Foundation (Leiden, The Netherlands). Consent for the use of residual donor material for research was obtained according to local legislation. Human exocrine cells were cultured as previously [21, 22].

xCT+/+ mice (WT) and xCT−/− mice (KO) are high-generation descendants of the strain originally described by Sato et al. [23]. Exocrine cells were isolated from mice as previously [24].

### Cell death assay

Cell death assay was performed with IncuCyte® Cytotox Green Reagent (4633, Essen Bioscience) according to the manufacturer’s instructions. A final concentration of 1:4000 dilution in culturing medium was used. Cells were imaged by IncuCyte Zoom System (Essen Bioscience) and EVOS M7000 auto digital microscope (Thermo Fisher Scientific) applying Z-stack.

### Mouse experiments

Female xCT+/+ and xCT−/− mice at 8–12 weeks age were treated with 125 µg/kg body weight caerulein (Eurogentec) according to a protocol adapted from Grimont et al. [25]. Control mice received saline. Mice were sacrificed 8 h after the first injection. The mice used were littermates without abnormalities and therefore not randomized, but subsequent sample processing and analysis were carried out in a blinded manner. Experiments were approved by the Ethical Committee for Animal Testing at the Vrije Universiteit Brussel (#21-637-1).

### Lipid peroxidation detection

The lipid peroxidation detection was performed with BODIPY™ 581/591 C11 probe (D3861, Invitrogen). To detect the accumulation of lipid peroxide, BODIPY was added to the medium at a final concentration of 5 μM. DNA staining was performed simultaneously using Hoechst (H1399, Invitrogen, 1/500). The cells were incubated for 20 min at 37 °C, followed by twice washing with HBSS (14-175-095, Gibco). Images were acquired using an EVOS M7000 auto digital microscope (Thermo Fisher Scientific).

### Glutathione detection

The glutathione detection was performed with the GSH-Glo™ Glutathione Assay (V6911, Promega). The glutathione detection on single cell level was normalized by the cell viability (using CellTiter-Glo G7571, Promega), measured by the GloMax Discover Microplate reader (Promega).

### Statistics

Sample sizes were determined based on the type of sample, being at least three independent biological repeats for cell lines, three to six biological repeats for mouse primary cells, four to five biological repeats for human primary cells, and three to five independent biological repeats for mouse in vivo experiments. No samples or animals were excluded from the analysis. Statistical analysis was performed using Prism v9.5 (GraphPad Software) applying paired or unpaired two-tailed parametric Student’s t-test, or one-way or two-way ANOVA with Tukey’s multiple comparisons tests. No significant variance difference was noted between the groups that were being compared. The number of independent repeats is indicated as N. ns: non-significant, *p < 0.05, **p < 0.01, ***p < 0.001, and ****p < 0.0001.

## Results

### xCT is transiently upregulated in human and mouse pancreatic acinar cells dedifferentiating under experimental stress conditions

**Fig. 1:**
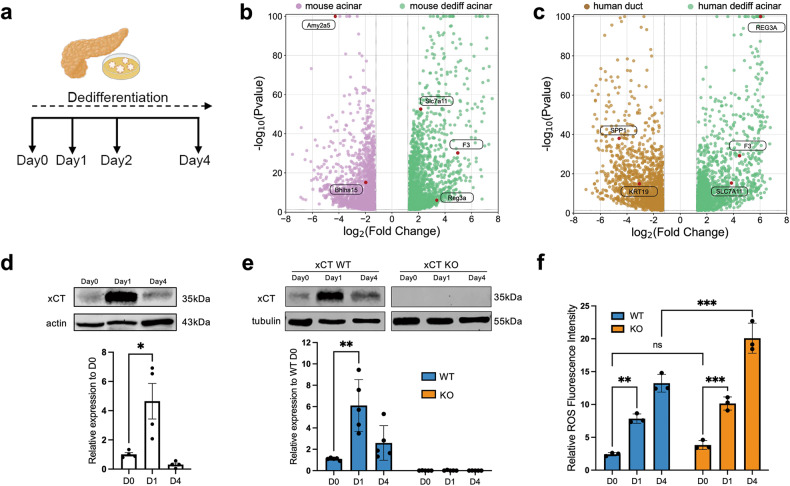
xCT is transiently upregulated in pancreatic dedifferentiating acinar cells. **a** Schematic representation of our in vitro model of human and mouse exocrine acinar and duct cells where acinar cells dedifferentiate and acquire ductal characteristics. **b** Volcano plot of most differentially expressed genes comparing mouse dedifferentiating exocrine cells at day 1 (right, green dots) and exocrine cells at day 0 (left, purple dots). Key genes were in red dots with name labels. **c** Volcano plot of most differentially expressed genes comparing human dedifferentiated acinar cells (right, green dots) and duct cells (left, brown dots). **d** Protein levels of xCT assessed by western blot in human exocrine cell cultures with quantification (Mean ± SD; *N* = 4; unpaired t-test). **e** Protein levels of xCT assessed by western blot in WT and KO mouse exocrine cell cultures with quantification (Mean ± SD; *N* = 5; unpaired t-test). **f** Relative ROS level assessed by Fluorometric Intracellular ROS Kit in WT/KO mouse exocrine cells at day 0 (D0), day 1 (D1), and day 4 (D4), respectively. Intensity represented in square root of normalized fluorometric reads (Mean ± SD; *N* = 3; two-way ANOVA with multiple comparisons).

While pancreatic acinar cells undergo dedifferentiation, they start to gain duct-like features. An experimental model recapitulating this process in vitro (with human or murine cells) was previously established in our lab [21, 22, 24] (Fig. 1a). When we profiled the transcriptome and compared the enriched pathways between mouse and human dedifferentiated acinar cells, we found multiple common pathways that were intertwined between cell differentiation and cell survival (Fig. S1a). “Focal adhesion” and “Cell adhesion molecules” are linked with acinar cluster formation [21, 24]. “MAPK signaling pathway”, “Hippo signaling pathway” and “Pl3K-Akt signaling pathway” are important pathways that can regulate acinar dedifferentiation and survival [26–30].

Our recent work already pointed out MECOM as specifically induced in dedifferentiated acinar cells allowing escape from apoptosis [20]. RNA sequencing of the mouse dedifferentiated acinar cells compared to their fully differentiated counterpart confirmed loss of acinar differentiation markers such as *Amy2a5*, while *Reg3a* and *F3* indicate ongoing dedifferentiation [21, 31]. This analysis revealed an upregulation of *Slc7a11* which codes for xCT that has known roles in stress response and cell survival (Fig. 1b). Moreover, in human cell cultures that were previously profiled [20], xCT was also specifically expressed in the dedifferentiated acinar cell population (together with *REG3A* and *F3* expression) and not in the duct cells of the same cultures (which are featured by ductal markers *KRT19* and *SPP1*) (Fig. 1c). When we then compared the most upregulated genes in mouse and human dedifferentiated acinar cells, xCT (*Slc7a11)* stood out from 14 genes in common (Supplementary Table 1, Fig. S1b). Hence, we monitored the expression of xCT in exocrine cell cultures from five individual human donor samples. All of them showed a transient increase of xCT mRNA and protein expression at the early timepoint of acinar cell dedifferentiation (day 1) with a further decline as ADM develops (Fig. S2a, Fig. 1d).

In a parallel experiment with isolated pancreatic acinar cells from healthy xCT+/+ mice (WT) and xCT−/− mice (KO), the results mimicked those of the human cells with a transient xCT induction (Fig. S2b, Fig. 1e). The KO cells confirmed the specificity of measuring xCT at mRNA and protein level (Fig. S2b, Fig. 1e). During dedifferentiation, we observed significant increases in intracellular ROS levels, with higher levels (at day 4) in KO compared to WT (Fig. 1f). However, while xCT expression was decreased at day 4, ROS levels remained high.

To summarize, acinar cells under stress conditions in vitro show a transient upregulation of xCT. This suggests a functional role of xCT in early stress response.

### Genetic deletion of xCT causes ferroptosis in murine acinar cells under stress

**Fig. 2:**
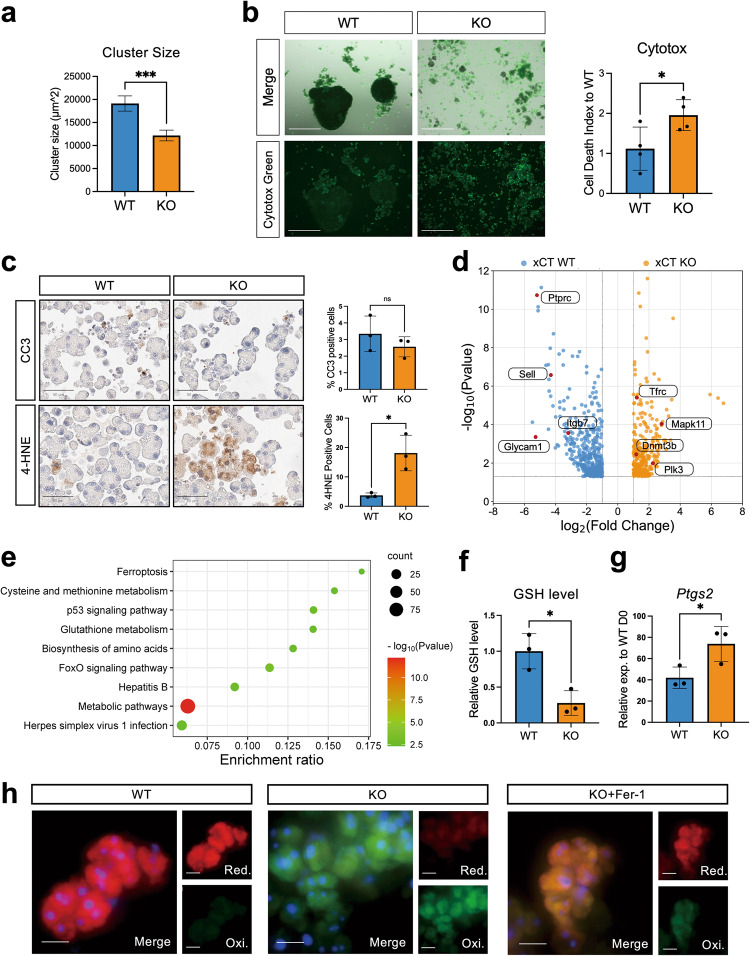
Genetic deletion of xCT causes ferroptosis in murine acinar cells under stress. **a** Quantification of cluster size of WT and xCT KO acinar cells (Mean ± SEM; *N* = 3; unpaired t-test). **b** Representative pictures with quantification of Cytotox Green (GFP) staining on xCT WT and KO mouse pancreatic acinar cells. Pictures were taken at day 4 of suspension culture by EVOS. The scale bar represents 600 µm. The cell death index was calculated by the ratio of GFP florescence intensity over cell viability and normalized to WT condition (Mean ± SD; *N* = 4; unpaired t-test). **c** Representative pictures of cleaved caspase 3 (CC3) and 4-Hydroxynonenal (4-HNE) adducts staining on WT and KO mouse acinar cell pellet sections at day 1 of suspension culture, respectively. The scale bar represents 50 μm. Quantification of each staining is shown next to the pictures (Mean ± SD; *N* = 3; unpaired t-test). **d** Volcano plot of most differentially expressed genes comparing xCT KO mouse acinar cells (right, orange dots) and xCT WT cells (left, blue dots) at day 0. **e** Bubble plot for pathways enriched with upregulated genes (*p* < 0.05) in KO cells at day 0 compared to WT. Pathway enrichment was performed and filtered by KOBAS software (*p* < 0.05). Count presents the number of genes enriched in a particular pathway. The enrichment ratio was calculated by the ratio of the number of genes enriched in a particular pathway to the total number of genes in this pathway. **f** Relative GSH level of WT and KO mouse acinar cells at day 1 of suspension culture (Mean ± SD; *N* = 3; unpaired t-test). **g** Relative mRNA expression of *Ptgs2* in WT and KO mouse acinar cells at day 1 of suspension culture (Mean ± S*D*; *N* = 3; unpaired t-test). **h** Representative pictures of C11-BODIPY staining for WT and KO acinar cells with or without Fer-1 treatment at day 1 of suspension culture. The red color represents reduced probe (Red.), indicating the basal level of lipid ROS. The green color represents oxidized form (Oxi.), indicating the accumulation of lipid ROS. The blue color is Hoechst dye which stains DNA. The scale bar represents 20 μm.

As shown in previous studies, a key feature of primary pancreatic acinar cells under stress in vitro is their capability of cluster formation which is crucial for their survival and further dedifferentiation [22, 25]. While acinar cells from WT mice could successfully aggregate into compact clusters by day 4 of culture, xCT KO cells showed a significant reduction in cluster size (Fig. 2a, Fig. S3a). Furthermore, there was more debris present in KO cultures, which confirmed by Cytotox Green was due to more cell death (Fig. 2b). Whereas early apoptosis has been reported during acinar cell dedifferentiation, including in this experimental model [1, 20], we did not observe any differences in the levels of apoptosis marker, cleaved caspase 3 (CC3) between the WT and KO genotypes (Fig. 2c).

We thus resorted to RNAseq analysis comparing WT and KO acinar clusters at the earliest timepoint after the cells had undergone the stress of the isolation process (Supplementary Table 1). For WT cells, genes such as *Itgb7*, *Glycam1*, *Sell*, and *Ptprc*, which are essential for cell adhesion were upregulated compared to KO cells (Fig. 2d) [32, 33]. This was in line with our observation of their larger cluster size (Fig. 2a). Meanwhile, 347 genes were highlighted to have higher expression in KO cells compared to WT. This KO gene set enriched for “Metabolic pathways” is suggestive of the metabolic changes in response to the genetic loss of the crucial amino acid transporter xCT (Fig. 2e). Interestingly, “Ferroptosis”, together with “Biosynthesis of amino acid”, “Glutathione metabolism”, and “Cysteine and methionine metabolism” appeared also in the top enriched pathways. These pathways reflected the scarcity of intracellular cysteine and the possible involvement of ferroptosis in our KO cells. xCT had indeed been extensively studied in the frame of ferroptosis [17, 18].

For assessing ferroptosis, the level of protein adducts as a byproduct of lipid peroxidation is measured by staining for 4-Hydroxynonenal (4-HNE). KO cells showed a significantly higher presence of 4-HNE (Fig. 2c). In addition, KO clusters showed a significantly lower GSH level suggesting increased vulnerability to oxidative stress (Fig. 2f). Ptgs2, although it can be induced in many inflammatory processes, has been recently found as a downstream marker for ferroptosis [34, 35]. We observed that *Ptgs2* was significantly upregulated in KO cells (Fig. 2g). As final confirmation of the involvement of ferroptosis, the C11-BODIPY probe that measures lipid peroxidation by staining cells at the basal state in red and turning into green when oxidized, consistently showed WT clusters in red and KO clusters in green due to the higher presence of lipid ROS (Fig. 2h). Treating xCT KO cells with Fer-1 (ferrostatin-1, a specific ferroptosis inhibitor) could indeed partially reverse lipid peroxide accumulation (Fig. 2h). Furthermore, Fer-1 could significantly rescue the reduced cluster size caused by xCT deletion (Fig. S3a, b).

Together these results indicate that loss of xCT in acinar cells under stress induces the depletion of intracellular cysteine, which further impairs ROS scavenging ability. As a consequence, lipid peroxide (lipid ROS) accumulates and triggers ferroptosis.

### Genetic knockdown of xCT causes ferroptosis in a pancreatic acinar cell line

**Fig. 3:**
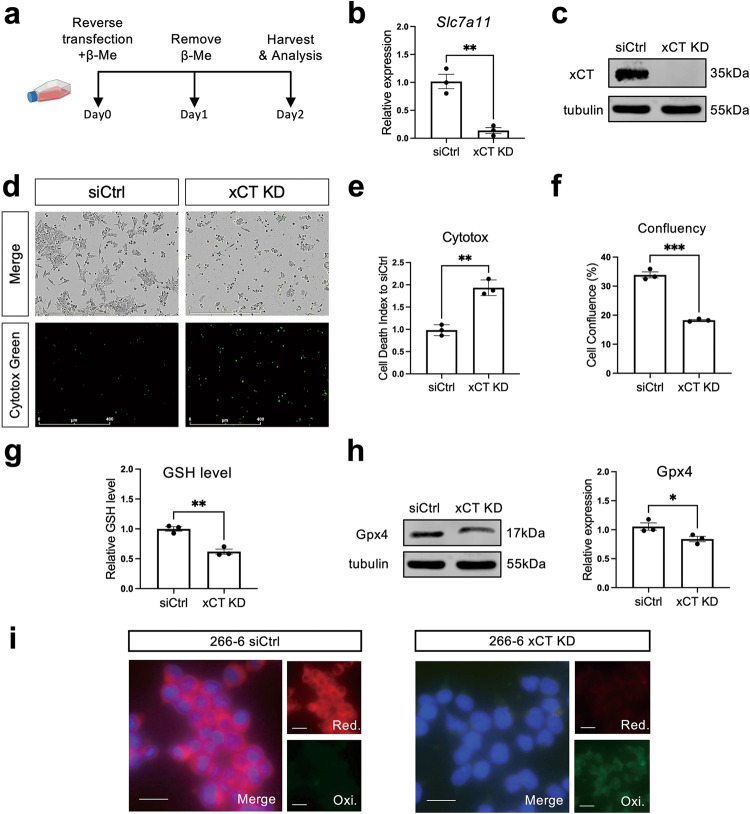
Genetic knockdown of xCT causes ferroptosis in a pancreatic acinar cell line. **a** Schematic demonstration of xCT knockdown (KD) mediated by siRNA in 266-6 cells. **b** mRNA levels of xCT assessed by qRT-PCR in 266-6 cells treated with scrambled siRNA (siCtrl) and xCT-siRNA (xCT KD) at day 2 after transfection (Mean ± SD; *N* = 3; unpaired t-test). **c** Protein levels of xCT assessed by western blot in siCtrl and xCT KD 266-6 cells at day 2 after transfection. **d** Representative pictures of phase contrast and Cytotox staining on siCtrl and xCT KD 266-6 cells, respectively. Pictures were taken at day 2 after transfection by IncuCyte. The scale bar represents 400 µm. **e** Quantification of cell death in siCtrl and xCT KD 266 cells. The cell death index was calculated by the ratio of Cytotox positive counts over cell confluency and normalized to siCtrl condtion (Mean ± SD; *N* = 3; unpaired t-test). **f** Quantification of cell confluency of siCtrl and xCT KD 266-6 cells at day 2 after transfection (Mean ± SD; *N* = 3; unpaired t-test). **g** Relative GSH level of siCtrl and xCT KD 266-6 cells at day 2 after transfection (Mean ± SD; *N* = 3; unpaired t-test). **h** Protein levels of Gpx4 with quantification assessed by western blot in siCtrl and xCT KD 266-6 cells at day 2 after transfection. **i** Representative pictures of C11-BODIPY staining of siCtrl and xCT KD 266-6 cells at day 2 after transfection. Red color represents reduced probe (Red.), indicating the basal level of lipid ROS. Green color represents oxidized form (Oxi.), indicating the accumulation of lipid ROS. Blue color is Hoechst dye which stains DNA. The scale bar represents 20 μm.

The mouse pancreatic acinar cell line 266-6 (CRL-2151) shows similarities to dedifferentiated acinar cells [20], including expression of xCT, and is thus a suitable model to study the consequence of xCT knockdown (KD). 2-Mercaptoethanol, which reduces cystine into cysteine, thus bypassing xCT [23], was added to the culture medium to maintain the viability of cells at the initial 24-h culture (Fig. 3a). Gene silencing for xCT was performed by transfection and knockdown was confirmed at mRNA and protein level (Fig. 3b, c).

xCT KD cells showed a different morphology and there were more single cells, which is less common for 266-6 cell cultures (Fig. 3d). The xCT KD cells compared to control (siCtrl) showed significantly higher cell death index and lower cell numbers (Fig. 3d–f). To assess the incidence of ferroptosis due to the lack of cystine transport in the xCT KD cells, we examined the xCT-GSH pathway. In line with the primary cell cultures above, intracellular GSH level was slightly yet significantly reduced in our xCT KD cells (Fig. 3g). Intracellular GSH relies on glutathione peroxidase 4 (GPX4) to counteract oxidative stress and Zhang et al. showed that cysteine depletion causes decreased GPX4 protein level [36], which we confirm in the xCT KD cells where Gpx4 protein expression was significantly lower (Fig. 3h). Hence, the availability of antioxidant, as well as the ability of ROS scavenging, was impaired at the same time. Indeed, xCT KD 266-6 cells showed a higher accumulation of lipid peroxidation, as demonstrated with the C11-BODIPY probe, and as a consequence, triggered ferroptosis (Fig. 3i). Intriguingly, Fer-1 failed to rescue xCT KD cells though (data not shown).

In conclusion, loss of xCT can lead to ferroptosis in a partially differentiated pancreatic acinar cell line. Consistently, xCT is crucial for maintaining GSH level, thus impacting the cell fate of dedifferentiated acinar cells by avoiding ferroptosis.

### Pharmacological inhibition of xCT leads to ferroptosis in a pancreatic acinar cell line

Since xCT genetic silencing induced ferroptosis in 266-6 cells, two pharmacological inhibitors were tested to see if they provoked similar effects. Sulfasalazine (SAS) is an FDA-approved pre-drug for rheumatoid arthritis and its activity of inhibiting xCT was discovered in recent years [37]. Erastin specifically binds to xCT and is widely known as an efficient xCT inhibitor and an inducer of ferroptosis [16].

**Fig. 4:**
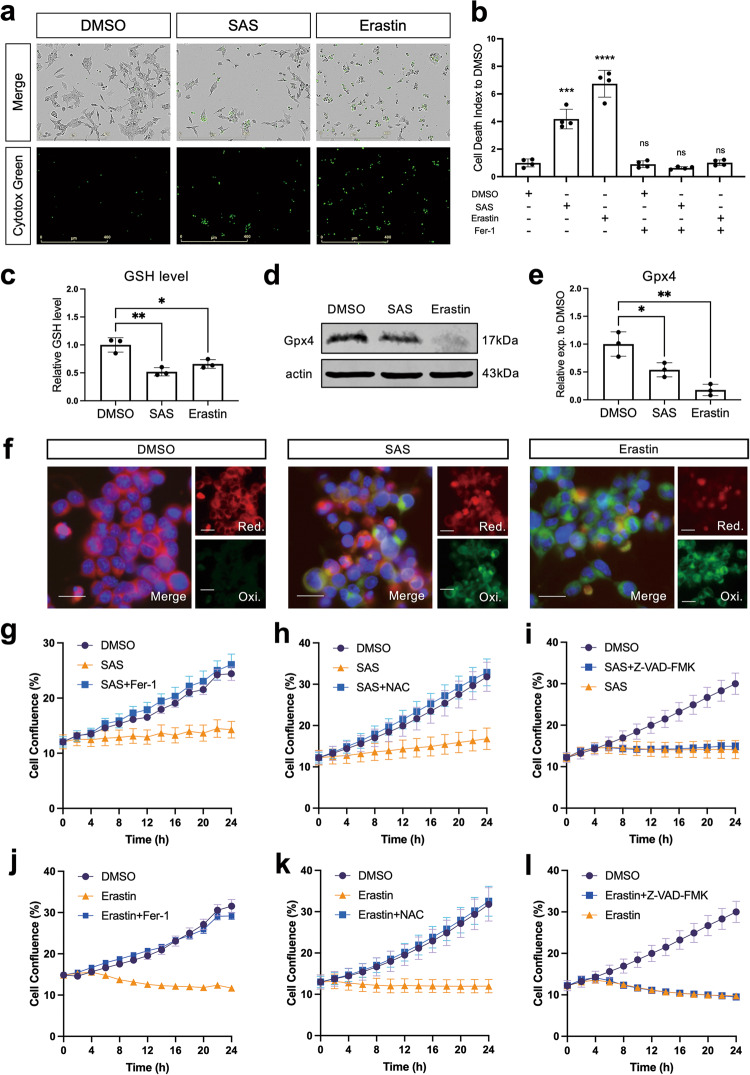
Pharmacological inhibition of xCT leads to ferroptosis in a pancreatic acinar cell line. **a** Representative IncuCyte pictures of phase contrast and Cytotox staining on 266-6 cells treated for 24 h with DMSO (0.05%), SAS (400 μM), or erastin (2.5 μM). The scale bar represents 400 µm. **b** Quantification of cell death in xCT inhibition and Fer-1 rescue conditions. The cell death index was calculated by the ratio of Cytotox positive counts over cell confluency and normalized to DMSO condition (Mean ± SD; *N* = 4; unpaired t-test compared to DMSO control). **c** Relative GSH levels of 266-6 cells in DMSO, SAS, and erastin conditions after 24 h of treatment (Mean ± SD; *N* = 3; unpaired t-test). **d**, **e** Protein levels of Gpx4 with quantification assessed by western blot in 266-6 cells in DMSO, SAS, and erastin conditions after 24 h of treatment (Mean ± SD; *N* = 3; unpaired t-test). **f** Representative pictures of C11-BODIPY staining of 266-6 cells in DMSO, SAS, and erastin conditions after 24 h of treatment. Red color represents reduced probe (Red.), indicating the basal level of lipid ROS. Green color represents oxidized form (Oxi.), indicating the accumulation of lipid ROS. Blue color is Hoechst dye which stains DNA. The scale bar represents 20 μm. **g**–**l** Cell confluency over time of 266-6 cells in DMSO, SAS, or erastin conditions rescued with or without Ferrostatin-1 (Fer-1), N-acetylcysteine (NAC) or Z-VAD-FMK, respectively (Mean ± SEM; *N* = 3).

After 24-h treatment of 266-6 cells with SAS or erastin, we observed significant increases of cell death and decreases of cell confluence (Fig. 4a, b and Fig. S3c for endpoint analysis, Fig. 4g–l for analysis over time). Similar to xCT KD cells, cells treated with xCT inhibitors showed reduced GSH level (Fig. 4c), decreased Gpx4 protein expression (Fig. 4d, e), and a predominant increase of lipid peroxidation (Fig. 4f). In almost every analysis, erastin effects were stronger than those of SAS. Erastin can trigger ferroptosis by targeting multiple molecules including xCT, anion channel and p53, thus making it a potent ferroptosis inducer [38, 39]. This may also explain why it showed stronger effect than xCT KD. The addition of ferroptosis inhibitor Fer-1 fully rescued the cell death and impaired cell confluency in presence of either of two xCT inhibitors (Fig. 4b, g, j). Moreover, introducing N-acetylcysteine (NAC), which functions as an antioxidant refilling the GSH level, also fully restored impaired cell number in both SAS and erastin-treated conditions (Fig. 4h, k) while the apoptosis inhibitor Z-VAD-FMK showed no rescue effect (Fig. 4i, l). Besides, we tested SAS or erastin in human exocrine suspension cultures and observed significant reductions in cluster size upon both xCT inhibitors treatment (Fig. S3d).

Taken together, pharmacological xCT inhibition can cause ferroptosis in the pancreatic acinar cell context making it a druggable target.

### xCT prevents lipid peroxidation in caerulein-induced acute pancreatitis in mice

**Fig. 5:**
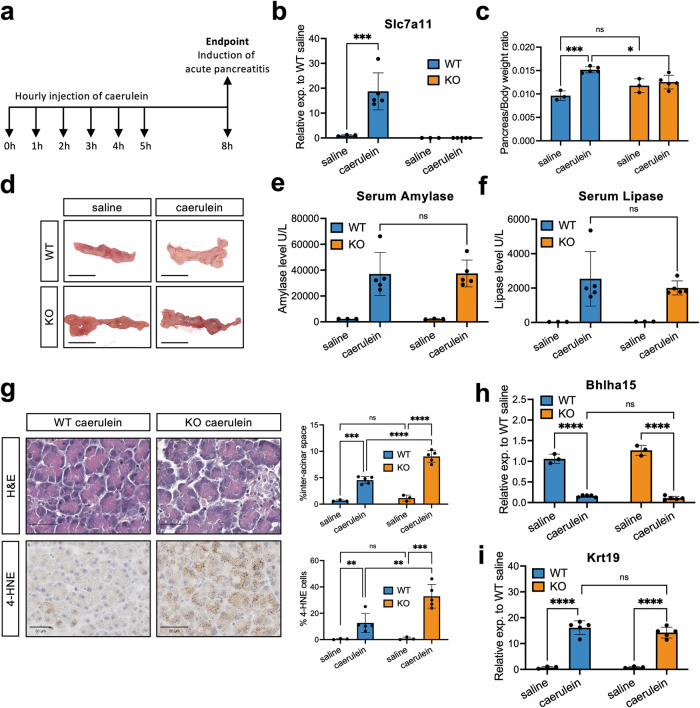
xCT prevents lipid peroxidation in caerulein-induced acute pancreatitis in mouse. **a** Schematic representation of injection of caerulein for inducing pancreatitis in mouse. **b** mRNA levels of xCT assessed by qRT-PCR in saline/caerulein treated xCT WT/KO mouse pancreatic tissue (Mean ± SD; each dot represents an individual mouse; for saline groups, *N* = 3; for caerulein groups, *N* = 5; two-way ANOVA with multiple comparisons). **c** Quantification of pancreas/body weight of saline/caerulein treated xCT WT/KO mice (Mean ± SD; *N* = 3 or 5; two-way ANOVA with multiple comparisons). **d** Representative pictures of the isolated mouse pancreas. The scale bar represents 1 cm. **e**, **f** Serum amylase and lipase levels in saline/caerulein treated xCT WT/KO mice, respectively (Mean ± SD; *N* = 3 or 5; two-way ANOVA with multiple comparisons). **g** Representative pictures of H&E staining and 4-HNE adducts staining. The scale bar represents 50 μm. Quantification of inter-acinar space percentage and quantification of 4-HNE positive cells in saline/caerulein treated xCT WT/KO mouse pancreatic tissue are shown next to the pictures (Mean ± SD; *N* = 3 or 5; two-way ANOVA with multiple comparisons). **h**, **i** mRNA levels of *Bhlha15* (Mist1) and *Krt19* assessed by qRT-PCR in saline/caerulein treated xCT WT/KO mouse pancreatic tissue (Mean ± SD; *N* = 3 or 5; two-way ANOVA with multiple comparisons).

Acinar cell dedifferentiation and ADM are typical events of acute pancreatitis [1, 22]. Acute pancreatitis can be experimentally modeled by repetitive injections of caerulein (Fig. 5a) [20, 25].

In line with the in vitro observations and assessment of public data (Fig. S4) [40], 6-h caerulein treatment significantly elevated xCT expression in WT mouse pancreas (Fig. 5b). While caerulein caused an increase in pancreas/body weight ratio in WT mice, xCT KO mice showed a macroscopically less swollen pancreas and no increase of this ratio (Fig. 5c, d). The swelling is likely due to edema, a typical feature of acute pancreatitis. Although the caerulein treatment effect was evident on serum amylase and lipase, there was no difference between the genotypes at this timepoint (Fig. 5e, f). At the histological level, effects are variable from lobe to lobe, but overall, more acellular inter-acinar spaces are present in the xCT KO mice suggesting cell shrinkage or cell death (Fig. 5g). More expression of 4-HNE in KO mice confirmed that xCT KO acinar cells were showing more lipid peroxidation (Fig. 5g). We also observed more presence of Acsl4 (Fig. S5a, b) and a trend of higher *Ptgs2* expression in KO tissues (Fig. S5c), supporting the increase of lipid peroxidation [41].

Besides, we compared the expression of acinar differentiation markers (*Bhlha15* and *Amy2*, encoding for Mist1 and amylase, respectively), and ductal differentiation markers (*Krt19* and *Sox9*, encoding keratin 19 and the transcription factor Sox9, respectively). None of the markers showed significant differences between genotypes (Fig. 5h, i and Fig. S5d, e).

In conclusion, in mouse acute pancreatitis, xCT plays a specific and critical role in attenuating lipid peroxidation of acinar cells that undergo dedifferentiation.

## Discussion

The strategy of pancreatic acinar cells to respond to stress consists of utilizing their widely reported plasticity and ability to dedifferentiate [2, 42]. Previously in our lab, we managed to identify a transcription factor (MECOM/*Evi-1*) that suppresses apoptosis during this process [20]. The present study further reveals that xCT, the functional unit of the cystine/glutamate antiporter system x_C_^-^, is upregulated typically in acinar cells thereby suppressing ferroptosis. Of note, xCT is only transiently upregulated and starts to decline after day 1. We presume reduced xCT might be due to the upregulated p53, which is known to negatively regulate xCT [43]. Indeed, Pinho et al. showed p53 protein upregulation over acinar culture [44], therefore xCT transcription may be repressed.

During the early phase of dedifferentiation, intracellular ROS levels increase together with xCT upregulation. However, as dedifferentiation further develops and xCT starts to decline, ROS levels remain high. Recent studies suggested ROS as an inducer of acinar cell dedifferentiation [45, 46]. Minati et al. who used the same model also showed elevated ROS levels on day 3 compared to day 1, which confirmed our observation [47]. To decipher whether mitochondrial ROS is involved when xCT is absent, we performed Seahorse ATP production rate assay. A similar metabolic phenotype of WT and KO dedifferentiating acinar cells is revealed (Fig. S2c–e), indicating that other compartments (e.g., cytoplasm, endoplasmic reticulum, peroxisome, etc.) rather than mitochondria might contribute to the higher trends of overall ROS in KO cells. It should be noted that it was technically impossible to measure mitochondrial ROS directly in our cell system though.

It was identified recently that ferroptosis is a form of programmed cell death distinct from apoptosis. Ferroptosis is characterized by the accumulation of iron and lipid peroxides, which leads to the destruction of cell membranes and eventual cell death [16, 17]. The link between xCT and ferroptosis has been widely studied in the context of cancer cells, including PDAC cells. With high RAS activation due to mutations that render KRAS constitutively active, xCT transcription can be promoted to meet the need for cancer cell homeostasis [13]. Daher et al. demonstrated that xCT ablation in PDAC cell lines induces ferroptosis via both nutrient and oxidative stress [48]. Badgley et al. showed that genetic deletion of xCT in mouse pancreas induces tumor-selective ferroptosis [14]. Where most studies of xCT in the pancreas thus focused on restraining tumor growth, our study provides insights that xCT is a critical element in non-cancer tissue homeostasis, more specifically in the protection of pancreatic acinar cells when experiencing stresses such as those in (experimental) pancreatitis. Also here, there is concurring activation of Ras signaling and, these are exactly the conditions known to favor pancreatic cancer initiation as the acinar cells dedifferentiate making them more susceptible to transformation [6, 9, 24, 27].

While other studies also started implicating ferroptosis as important in pancreatitis/ADM in mouse models [49–51], we here also add evidence with human pancreatic acinar cells, underscoring the relevance of the observations in patients. Thus far it is the only way to gain insights into these critical very early events in human pancreatitis.

Ferrostatin-1, as a ferroptosis inhibitor, is believed to function as a radical-trapping antioxidant that counteracts lipid ROS [52]. However, in ferroptotic cells, lipid hydroperoxides can also accumulate via lipoxygenase, on which Fer-1 has little inhibition potency [52]. This may explain why Fer-1 only showed a partial rescuing effect on mouse dedifferentiating acinar cells. Interestingly, Badgley et al. showed that Fer-1 could significantly but also only partially rescue lipid peroxidation in human PDAC cell lines [14]. While Fer-1 fully rescued cell death in xCT pharmacologically inhibited 266-6 cells, we also observed that Fer-1 failed to rescue our xCT KD cells. This suggests that genetic depletion and pharmacological inhibitions of xCT may vary in terms of triggering ferroptosis. Sato et al. found similar results that Lip-1 could only rescue lipid peroxidation but not cell cycle arrest in xCT KO melanoma cells [53]. Hu et al. discovered that inhibition of xCT in lung cancer cells, despite decreasing GSH level and increasing ROS, did not necessarily lead to ferroptosis, but instead resulted in apoptosis [54]. On the one hand, we propose that complete pathway validation, rather than solely individual measurements such as lipid peroxidation (which is also elevated in non-canonical pyroptosis [55]) should be regarded for calling “ferroptosis”. On the other hand, we should keep vigilant in comparing pharmacological inhibitors versus xCT genetic KO/KD models, next to the possible differences in cell types, mutational context, etc.

We observed that while the pancreas from xCT KO mice did not show obvious abnormalities compared to WT in the healthy condition, the external stress caused by caerulein led to more lipid peroxidation in the xCT KO acinar cells. Whereas WT and KO mice both showed typical features of pancreatitis (e.g., serum amylase/lipase or gene expression changes in acinar and ductal genes), WT mice showed more swollen pancreas which might be due to edema (Fig. 5d). Since macrophage is one of the main sources of cytokines (e.g., IL-1β) during early inflammation, and xCT is associated with cytokine changes and macrophage function [56–58], less edema in KO pancreas could possibly be explained by less or delayed macrophage infiltration. Nevertheless, the pancreas did not reduce in size, and at the cellular level, cell differentiation markers did not differ between the xCT KO cells and their WT counterparts (Fig. 5h, i and Fig. S5d–g). One reason for less explicit phenotypes in vivo than in vitro is the fact that different lobes of the pancreas respond heterogeneously to caerulein, which is often seen but underreported [25, 59]. This may mask lobe-specific events in a context of huge changes in gene/protein expression. Nevertheless, we believe that these findings uncouple the processes of cell survival and differentiation with a prominent role of xCT specifically in the former.

We also realize that there is a lower dependency on xCT as ADM further develops since we observed that xCT expression was much lower in human ductal cells than acinar cells early in their dedifferentiation process, and there is a declining enrichment score of ferroptosis when ADM progresses (Fig. S1c). This further suggests that the survival response is a critical gatekeeper. When overcoming ferroptosis, acinar cells can dedifferentiate. More elegant experimental models, including inducible models that also allow assessment at the single cell level, should be designed to decipher these stages in detail. Such future studies should also take into account heterogenous subsets of acinar cell populations [60]. In addition, intrinsic compensatory mechanisms can often be noted in constitutive KO models. For example, the presence of intercellular cysteine shuttle between neighboring cells could provide some protection against ferroptosis in the xCT KO mice [61]. It is also worth noticing that genes involved in FoxO signaling pathway (such as *Plk3* and *Mapk11*) were highly expressed in xCT KO cells (Fig. 2d, e). Considering the oxidative stress resistance function of FoxO signaling pathway, this may provide hints for possible compensatory mechanisms [62, 63].

Inhibiting xCT has already shown great potential for cancer treatment. Inactivating xCT with ferroptosis inducers sensitized the cells for radiotherapy and immunotherapy and therefore open paths to treatment [64–66]. We here show that xCT inhibitors such as SAS or erastin can force acinar cells under stress into cell death, instead of dedifferentiating. One could envision xCT being expressed at the tipping point of cell fate decisions and offering a therapeutic intervention strategy for halting tumor development in high-risk individuals, e.g., those suffering from recurrent acute or chronic pancreatitis. The data of Badgley et al. [14] showed indeed that fewer tumors develop in absence of xCT. However, it remains unclear if this is the role of xCT transiently expressed during the early stress and Ras activation or by the absence of xCT in the eventual tumor that also relies on it. Aforementioned targeting of xCT as a tumor prevention strategy confers a narrow window of opportunity when it comes to xCT early and transiently being expressed in the initial stress, but xCT expression remains expressed in the tumor [13, 14]. Another hurdle to overcome is the fact that the current xCT inhibitors lack specificity and stability, and of the new inhibitor HG106 (that we did not test here), the mechanism of action requires more investigation [54]. While several studies have suggested that redifferentiation (reversal of ADM) or maintenance of acinar characteristics showed benefits to overcome pancreatitis, and thus could be a therapeutic intervention to stop tumor development [47, 67, 68], we add an option to selectively push dedifferentiated acinar cells into ferroptosis. In summary, the present study has revealed novel fundamental insights into pancreatic acinar cell fate when under stress, relevant for major diseases of the exocrine pancreas.

## Supplementary information

Supplementary Data

Supplementary Table 1

Original Data File

Reproducibility checklist

## Data Availability

RNA-seq data have been deposited in Gene Expression Omnibus (GEO) and are accessible through GEO Series accession number GSE240106. Additional data are available from the corresponding author on reasonable request.

## References

[1] Rooman I, Real FX (2012). Pancreatic ductal adenocarcinoma and acinar cells: a matter of differentiation and development?. Gut.

[2] Backx E, Coolens K, Van den Bossche JL, Houbracken I, Espinet E, Rooman I (2022). On the origin of pancreatic cancer: molecular tumor subtypes in perspective of exocrine cell plasticity. Cell Mol Gastroenterol Hepatol..

[3] Storz P (2017). Acinar cell plasticity and development of pancreatic ductal adenocarcinoma HHS Public Access. Nat Rev Gastroenterol Hepatol..

[4] Eser S, Reiff N, Messer M, Seidler B, Gottschalk K, Dobler M (2013). Selective requirement of PI3K/PDK1 signaling for Kras oncogene-driven pancreatic cell plasticity and cancer. Cancer Cell..

[5] Storz P, Crawford HC (2020). Carcinogenesis of pancreatic ductal adenocarcinoma. Gastroenterology.

[6] Assi M, Achouri Y, Loriot A, Dauguet N, Dahou H, Baldan J (2021). Dynamic regulation of expression of KRAS and its effectors determines the ability to initiate tumorigenesis in pancreatic acinar cells. Cancer Res..

[7] Krah NM, De La OJP, Swift GH, Hoang CQ, Willet SG, Chen Pan F (2015). The acinar differentiation determinant PTF1A inhibits initiation of pancreatic ductal adenocarcinoma. Elife.

[8] Von Figura G, Morris JP, Wright CVE, Hebrok M (2014). Nr5a2 maintains acinar cell differentiation and constrains oncogenic Kras-mediated pancreatic neoplastic initiation. Gut.

[9] Logsdon CD, Ji B (2009). Ras activity in acinar cells links chronic pancreatitis and pancreatic cancer. Clin Gastroenterol Hepatol..

[10] Roy N, Takeuchi KK, Ruggeri JM, Bailey P, Chang D, Li J (2016). PDX1 dynamically regulates pancreatic ductal adenocarcinoma initiation and maintenance. Genes Dev..

[11] Meyer AR, Engevik AC, Willet SG, Williams JA, Zou Y, Massion PP (2019). Cystine/glutamate antiporter (xCT) is required for chief cell plasticity after gastric injury. Cell Mol Gastroenterol Hepatol.

[12] Koppula P, Zhang Y, Zhuang L, Gan B (2018). Amino acid transporter SLC7A11/xCT at the crossroads of regulating redox homeostasis and nutrient dependency of cancer. Cancer Commun..

[13] Lim JKM, Delaidelli A, Minaker SW, Zhang HF, Colovic M, Yang H (2019). Cystine/glutamate antiporter xCT (SLC7A11) facilitates oncogenic RAS transformation by preserving intracellular redox balance. Proc Natl Acad Sci USA..

[14] Badgley MA, Kremer DM, Carlo Maurer H, DelGiorno KE, Lee HJ, Purohit V (2020). Cysteine depletion induces pancreatic tumor ferroptosis in mice. Science.

[15] Zhang W, Sun Y, Bai L, Zhi L, Yang Y, Zhao Q (2021). RBMS1 regulates lung cancer ferroptosis through translational control of SLC7A11. J Clin Invest..

[16] Dixon SJ, Lemberg KM, Lamprecht MR, Skouta R, Zaitsev EM, Gleason CE (2012). Ferroptosis: an iron-dependent form of nonapoptotic cell death. Cell.

[17] Li J, Cao F, Yin H-L, Huang Z-J, Lin Z-T, Mao N (2020). Ferroptosis: past, present and future. Cell Death Dis..

[18] Jyotsana N, Ta KT, DelGiorno KE (2022). The role of cystine/glutamate antiporter SLC7A11/xCT in the pathophysiology of cancer. Front Oncol.

[19] Chuvin N, Vincent DF, Pommier RM, Alcaraz LB, Gout J, Caligaris C (2017). Acinar-to-ductal metaplasia induced by transforming growth factor beta facilitates KRASG12D-driven pancreatic tumorigenesis. Cell Mol Gastroenterol Hepatol..

[20] Backx E, Wauters E, Baldan J, Van Bulck M, Michiels E, Heremans Y (2021). MECOM permits pancreatic acinar cell dedifferentiation avoiding cell death under stress conditions. Cell Death Differ..

[21] Baldan J, Houbracken I, Rooman I, Bouwens L (2019). Adult human pancreatic acinar cells dedifferentiate into an embryonic progenitor-like state in 3D suspension culture. Sci Rep..

[22] Houbracken I, De Waele E, Lardon J, Ling Z, Heimberg H, Rooman I (2011). Lineage tracing evidence for transdifferentiation of acinar to duct cells and plasticity of human pancreas. Gastroenterology.

[23] Sato H, Shiiya A, Kimata M, Maebara K, Tamba M, Sakakura Y (2005). Redox imbalance in cystine/glutamate transporter-deficient mice. J Biol Chem..

[24] Pinho AV, Rooman I, Reichert M, De Medts N, Bouwens L, Rustgi AK (2011). Adult pancreatic acinar cells dedifferentiate to an embryonic progenitor phenotype with concomitant activation of a senescence programme that is present in chronic pancreatitis. Gut.

[25] Grimont A, Pinho AV, Cowley MJ, Augereau C, Mawson A, Giry-Laterrière M (2015). SOX9 regulates ERBB signalling in pancreatic cancer development. Gut.

[26] Wu Y, Aegerter P, Nipper M, Ramjit L, Liu J, Wang P (2021). Hippo signaling pathway in pancreas development. Front Cell Dev Biol..

[27] Baer R, Cintas C, Dufresne M, Cassant-Sourdy S, Schönhuber N, Planque L (2014). Pancreatic cell plasticity and cancer initiation induced by oncogenic Kras is completely dependent on wild-type PI 3-kinase p110α. Genes Dev..

[28] Collins MA, Yan W, Sebolt-Leopold JS, Pasca Di Magliano M (2014). MAPK signaling is required for dedifferentiation of acinar cells and development of pancreatic intraepithelial neoplasia in mice. Gastroenterology.

[29] Tamura T, Kodama T, Sato K, Murai K, Yoshioka T, Shigekawa M (2021). Dysregulation of PI3K and Hippo signaling pathways synergistically induces chronic pancreatitis via CTGF upregulation. J Clin Invest..

[30] Lin R, Bao X, Wang H, Zhu S, Liu Z, Chen Q (2021). TRPM2 promotes pancreatic cancer by PKC/MAPK pathway. Cell Death Dis.

[31] Li Q, Wang H, Zogopoulos G, Shao Q, Dong K, Lv F (2016). Reg proteins promote acinar-to-ductal metaplasia and act as novel diagnostic and prognostic markers in pancreatic ductal adenocarcinoma. Oncotarget.

[32] Neri P, Ren L, Azab AK, Brentnall M, Gratton K, Klimowicz AC (2011). Integrin β7-mediated regulation of multiple myeloma cell adhesion, migration, and invasion. Blood.

[33] Wang A, Chen M, Wang H, Huang J, Bao Y, Gan X (2019). Cell adhesion-related molecules play a key role in renal cancer progression by multinetwork analysis. Biomed Res Int..

[34] Yang WS, SriRamaratnam R, Welsch ME, Shimada K, Skouta R, Viswanathan VS (2014). Regulation of ferroptotic cancer cell death by GPX4. Cell.

[35] Ethridge RT, Chung DH, Slogoff M, Ehlers RA, Hellmich MR, Rajaraman S (2002). Cyclooxygenase-2 gene disruption attenuates the severity of acute pancreatitis and pancreatitis-associated lung injury. Gastroenterology.

[36] Zhang Y, Swanda RV, Nie L, Liu X, Wang C, Lee H (2021). mTORC1 couples cyst(e)ine availability with GPX4 protein synthesis and ferroptosis regulation. Nat Commun..

[37] Gout PW, Buckley AR, Simms CR, Bruchovsky N (2001). Sulfasalazine, a potent suppressor of lymphoma growth by inhibition of the x(c)- cystine transporter: a new action for an old drug. Leukemia.

[38] Zhao Y, Li Y, Zhang R, Wang F, Wang T, Jiao Y (2020). The role of erastin in ferroptosis and its prospects in cancer therapy. Onco Targets Ther..

[39] Sato M, Kusumi R, Hamashima S, Kobayashi S, Sasaki S, Komiyama Y (2018). The ferroptosis inducer erastin irreversibly inhibits system xc- and synergizes with cisplatin to increase cisplatin’s cytotoxicity in cancer cells. Sci Rep..

[40] Cobo I, Martinelli P, Flández M, Bakiri L, Zhang M, Carrillo-De-Santa-Pau E (2018). Transcriptional regulation by NR5A2 links differentiation and inflammation in the pancreas. Nature.

[41] Yuan H, Li X, Zhang X, Kang R, Tang D (2016). Identification of ACSL4 as a biomarker and contributor of ferroptosis. Biochem Biophys Res Commun..

[42] Storz P (2017). Acinar cell plasticity and development of pancreatic ductal adenocarcinoma. Nat Rev Gastroenterol Hepatol..

[43] Jiang L, Kon N, Li T, Wang SJ, Su T, Hibshoosh H (2015). Ferroptosis as a p53-mediated activity during tumour suppression. Nature.

[44] Pinho AV, Rooman I, Real FX (2011). p53-dependent regulation of growth, epithelial-mesenchymal transition and stemness in normal pancreatic epithelial cells. Cell Cycle.

[45] Döppler HR, Liou GY, Storz P (2022). Generation of hydrogen peroxide and downstream protein kinase D1 signaling is a common feature of inducers of pancreatic acinar-to-ductal metaplasia. Antioxidants.

[46] Liou GY, Döppler H, DelGiorno KE, Zhang L, Leitges M, Crawford HC (2016). Mutant KRas-induced mitochondrial oxidative stress in acinar cells upregulates EGFR signaling to drive formation of pancreatic precancerous lesions. Cell Rep..

[47] Minati MA, Libert M, Dahou H, Jacquemin P, Assi M (2021). N-acetylcysteine reduces the pro-oxidant and inflammatory responses during pancreatitis and pancreas tumorigenesis. Antioxidants.

[48] Daher B, Parks SK, Durivault J, Cormerais Y, Baidarjad H, Tambutte E (2019). Genetic ablation of the cystine transporter xCT in PDAC cells inhibits mTORC1, growth, survival, and tumor formation via nutrient and oxidative stresses. Cancer Res..

[49] Ma X, Dong X, Xu Y, Ma N, Wei M, Xie X (2023). Identification of AP-1 as a critical regulator of glutathione Peroxidase 4 (GPX4) transcriptional suppression and acinar cell ferroptosis in acute pancreatitis. Antioxidants.

[50] Tian C, Zhao J, Xiong Q, Yu H, Du H (2023). Secondary iron overload induces chronic pancreatitis and ferroptosis of acinar cells in mice. Int J Mol Med..

[51] Liu K, Liu J, Zou B, Li C, Zeh HJ, Kang R (2022). Trypsin-mediated sensitization to ferroptosis increases the severity of pancreatitis in mice. CMGH.

[52] Zilka O, Shah R, Li B, Friedmann Angeli JP, Griesser M, Conrad M (2017). On the mechanism of cytoprotection by ferrostatin-1 and liproxstatin-1 and the role of lipid peroxidation in ferroptotic cell death. ACS Cent Sci..

[53] Sato M, Onuma K, Domon M, Hasegawa S, Suzuki A, Kusumi R (2020). Loss of the cystine/glutamate antiporter in melanoma abrogates tumor metastasis and markedly increases survival rates of mice. Int J Cancer.

[54] Hu K, Li K, Lv J, Feng J, Chen J, Wu H (2020). Suppression of the SLC7A11/glutathione axis causes synthetic lethality in KRAS-mutant lung adenocarcinoma. J Clin Invest..

[55] Wiernicki B, Dubois H, Tyurina YY, Hassannia B, Bayir H, Kagan VE (2020). Excessive phospholipid peroxidation distinguishes ferroptosis from other cell death modes including pyroptosis. Cell Death Dis.

[56] Hu F, Lou N, Jiao J, Guo F, Xiang H, Shang D (2020). Macrophages in pancreatitis: mechanisms and therapeutic potential. Biomed Pharmacother..

[57] Albertini G, Deneyer L, Ottestad-Hansen S, Zhou Y, Ates G, Walrave L (2018). Genetic deletion of xCT attenuates peripheral and central inflammation and mitigates LPS-induced sickness and depressive-like behavior in mice. Glia.

[58] Pfau JC, Seib T, Overocker JJ, Roe J, Ferro AS (2012). Functional expression of system x(c)- is upregulated by asbestos but not crystalline silica in murine macrophages. Inhal Toxicol..

[59] Pandol SJ, Saluja AK, Imrie CW, Banks PA (2007). Acute pancreatitis: bench to the bedside. Gastroenterology.

[60] Schlesinger Y, Yosefov-Levi O, Kolodkin-Gal D, Granit RZ, Peters L, Kalifa R (2020). Single-cell transcriptomes of pancreatic preinvasive lesions and cancer reveal acinar metaplastic cells’ heterogeneity. Nat Commun..

[61] Meira W, Daher B, Parks SK, Cormerais Y, Durivault J, Tambutte E (2021). A cystine-cysteine intercellular shuttle prevents ferroptosis in xctko pancreatic ductal adenocarcinoma cells. Cancers.

[62] Glauser DA, Schlegel W (2007). The emerging role of FOXO transcription factors in pancreatic beta cells. J Endocrinol..

[63] Klotz LO, Sánchez-Ramos C, Prieto-Arroyo I, Urbánek P, Steinbrenner H, Monsalve M (2015). Redox regulation of FoxO transcription factors. Redox Biol..

[64] Lei G, Zhang Y, Koppula P, Liu X, Zhang J, Lin SH (2020). The role of ferroptosis in ionizing radiation-induced cell death and tumor suppression. Cell Res..

[65] Ye LF, Chaudhary KR, Zandkarimi F, Harken AD, Kinslow CJ, Upadhyayula PS (2020). Radiation-induced lipid peroxidation triggers ferroptosis and synergizes with ferroptosis inducers. ACS Chem Biol.

[66] Lang X, Green MD, Wang W, Yu J, Choi JE, Jiang L (2019). Radiotherapy and immunotherapy promote tumoral lipid oxidation and ferroptosis via synergistic repression of SLC7A11. Cancer Discov..

[67] Krah NM, Narayanan SM, Yugawa DE, Straley JA, Wright CVE, MacDonald RJ (2019). Prevention and reversion of pancreatic tumorigenesis through a differentiation-based mechanism. Dev Cell.

[68] Murtaugh LC, Keefe MD (2015). Regeneration and repair of the exocrine pancreas. Annu Rev Physiol..

