# Supplementary material for: Pancreatic acinar cell fate relies on system xC- to prevent ferroptosis during stress
Source: Cell Death Dis. 2023 Aug 21;14(8):536. doi: 10.1038/s41419-023-06063-w (PMC10442358; doi:10.1038/s41419-023-06063-w)
Supplement: Supplementary file 1 — Supplementary Data [file 41419_2023_6063_MOESM1_ESM.docx]

**SUPPLEMENTARY DATA**

**Supplementary Table**. RNAseq data of differentially expressed genes. Comparison 1: WT mouse acinar cells (day 1 vs day 0). Comparison 2: mouse acinar cells at day 0 (WT vs KO).

**Supplementary Figure 1. RNAseq analysis - KEGG pathways enriched in human and mouse pancreatic (de-)differentiation acinar cells.** (a) Common KEGG pathways enriched in both human and mouse dedifferentiated exocrine cell suspension cultures compared to their differentiated population respectively. (b) Venn diagram of most upregulated common genes (Log Foldchange >2.5, P-value <0.05) in mouse and human dedifferentiated acinar cells compared to their differentiated counterpart. (c) Upregulated KEGG pathways enriched in xCT KO mouse acinar cell suspension culture compared to corresponding WT populations over time.

**
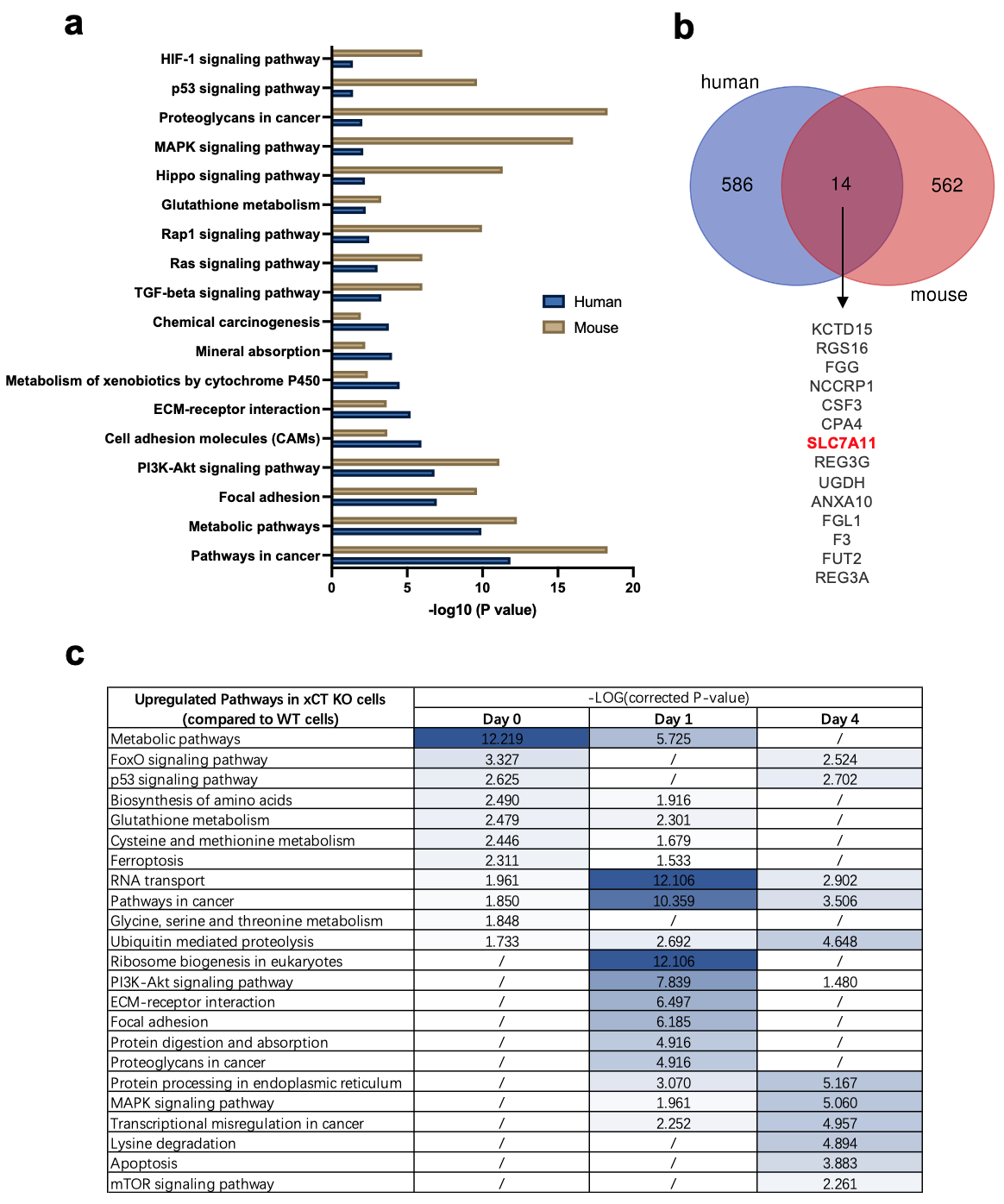
**

**Supplementary Figure 2. xCT expression and Seahorse analysis.** (a) mRNA levels of xCT assessed by qRT-PCR in human exocrine cell cultures (N=6). (b) mRNA levels of xCT assessed by qRT-PCR in WT and KO mouse exocrine cell cultures (Mean±SD; N=5; unpaired t-test). Genes with Ct values above 35 were regarded as no expression. (c-d) Oxygen consumption rate and proton efflux rate of WT and KO mouse dedifferentiating acinar cells at day 1 (Mean±SD; N=6). (e) ATP production rate from mitochondrial respiration or glycolysis in WT and KO mouse dedifferentiating acinar cells at day 1 (Mean±SD; N=6; unpaired t-test).


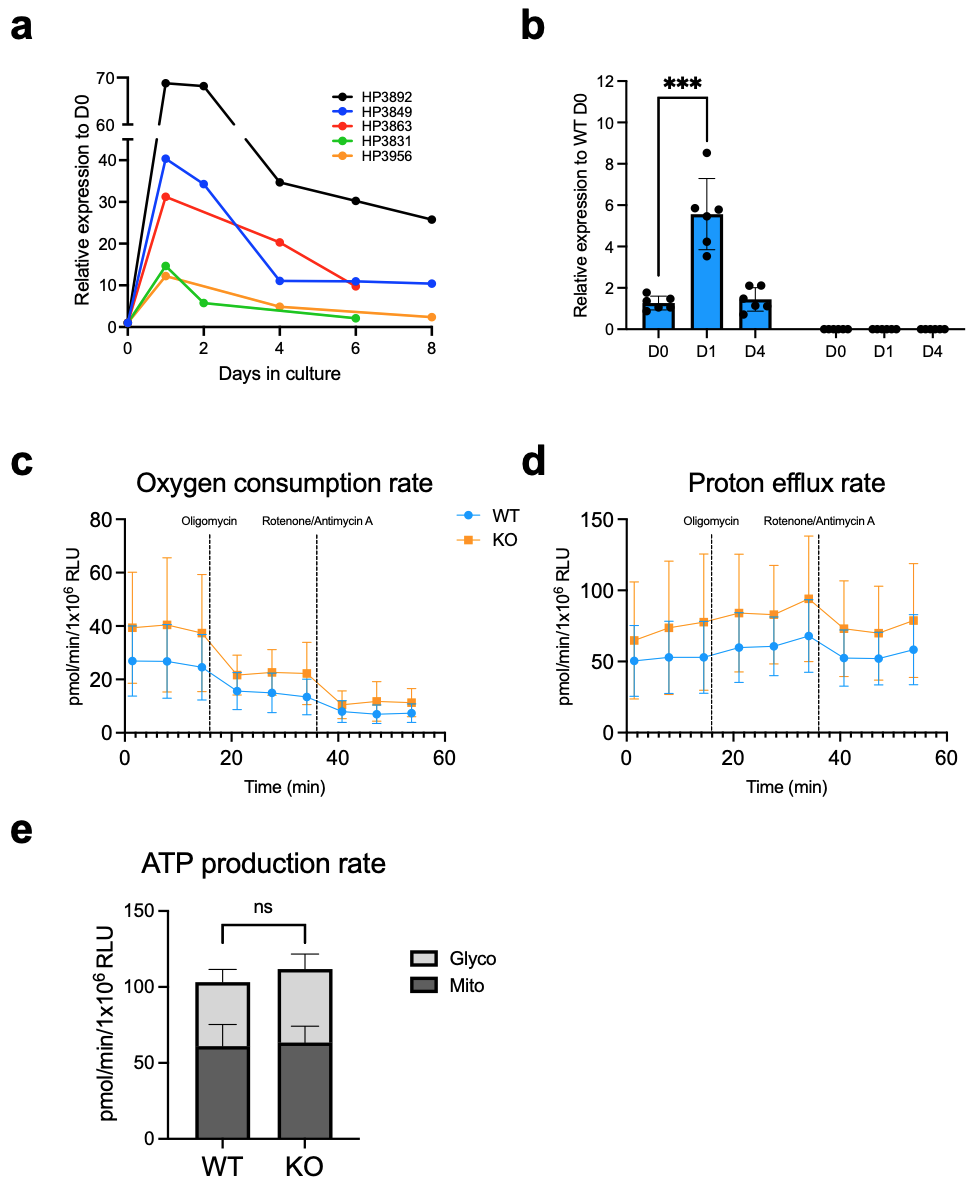


**Supplementary Figure 3. Cluster size measurement and xCT inhibition.** (a) Representative pictures of clusters of xCT WT mouse acinar cells, KO cells, and KO cells treated with Fer-1. Pictures were taken on day 4 of suspension culture. The scale bar represents 500μm. (b) Quantification of cluster size (Mean±SEM; N=3; two-way ANOVA with multiple comparisons). (c) Quantification of cell confluency in DMSO, SAS and erastin conditions after 24 hours of treatment (Mean±SD; N=3; unpaired t-test). (d) Quantification of cluster size of human exocrine cultures treated with DMSO (0.05%), SAS (400μM) or erastin (2.5μM). Pictures were taken at day 4 of suspension culture. (Mean±SEM; N=2; two-way anova with multiple comparisons).

**
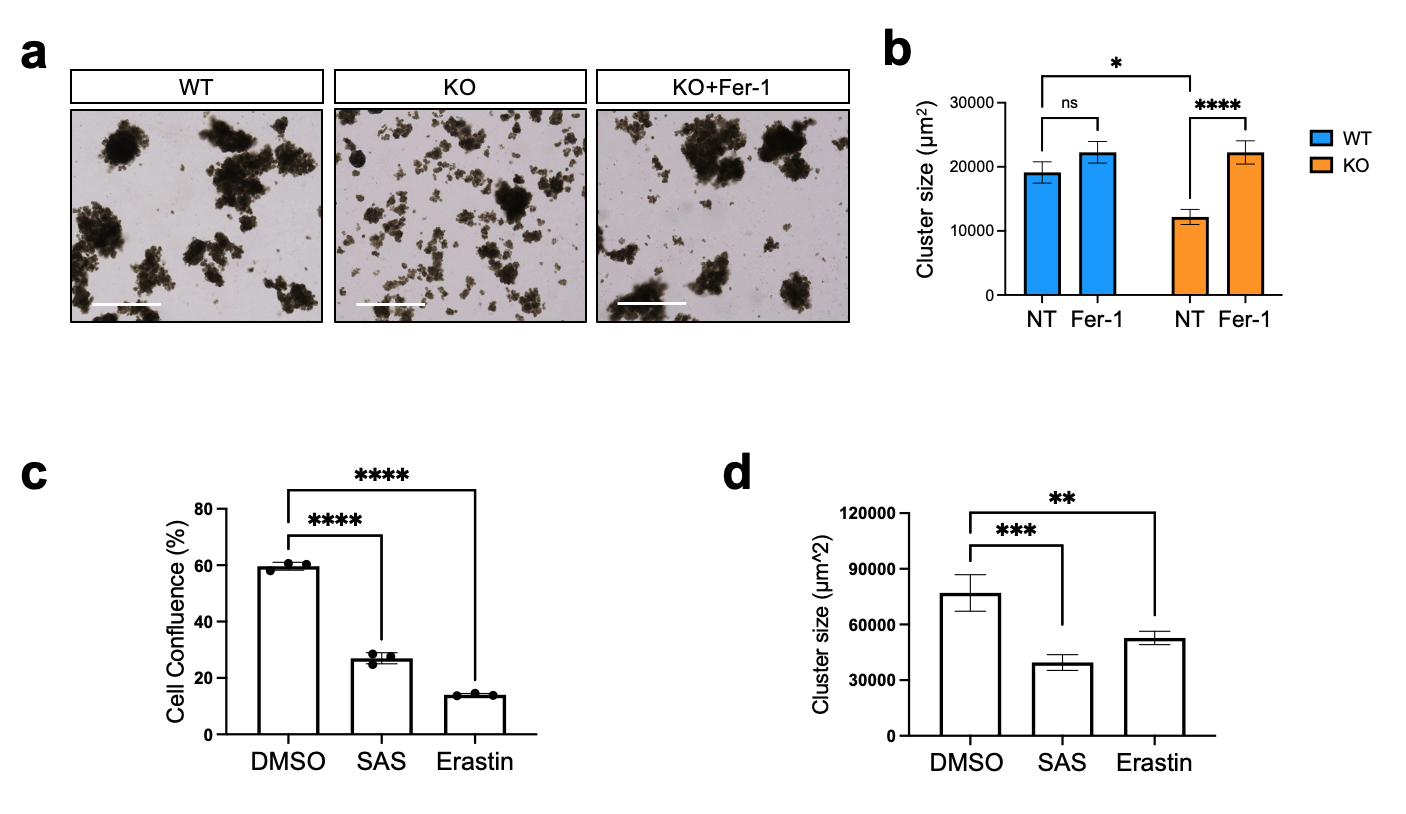
**

**Supplementary Figure 4. Evaluation of xCT and dedifferentiation markers in a caerulein model of pancreatitis (publicly available data from Cobo et al. 2018)** (a) mRNA levels of xCT assessed by RNAseq in saline/caerulein treated WT mouse pancreatic tissue (Mean±SD; N=3; unpaired t-test). (b) mRNA levels of *Amy2* assessed by RNAseq in saline/caerulein treated WT mouse pancreatic tissue (Mean±SD; N=3; unpaired t-test). (c) mRNA levels of *Krt19* assessed by RNAseq in saline/caerulein treated WT mouse pancreatic tissue (Mean±SD; N=3; unpaired t-test).

**
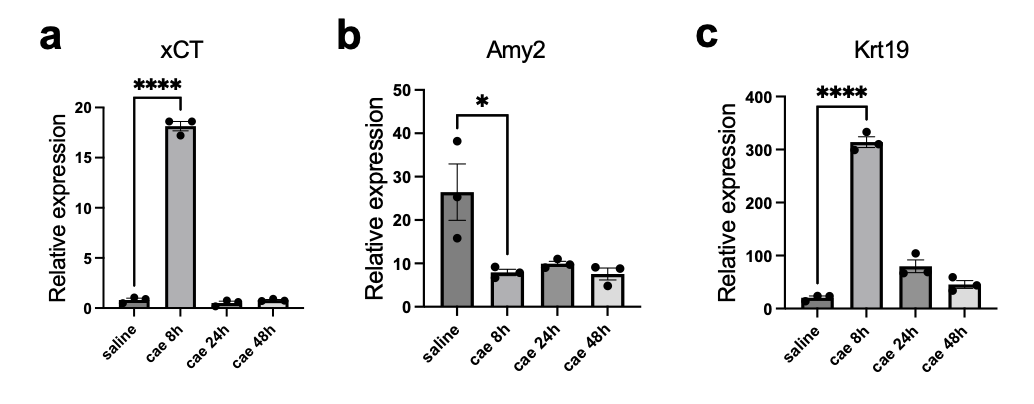
**

**Supplementary Figure 5. Acinar dedifferentiation and ferroptosis in mouse pancreatitis.** (a) Representative pictures of Acsl4 staining on saline/caerulein treated xCT WT/KO mouse pancreatic tissue. The scale bar represents 60µm. (b) Quantification of Acsl4 (represented in square root of percentage of positive cells) is shown next to the pictures (Mean±SD; N=3 or 5; two-way ANOVA with multiple comparisons). (c) mRNA levels of Ptgs2 assessed by qRT-PCR in saline/caerulein treated xCT WT/KO mouse pancreatic tissue (Mean±SD; N=3 or 5; two-way ANOVA with multiple comparisons). (d) mRNA levels of *Amy2* assessed by qRT-PCR in saline/caerulein treated xCT WT/KO mouse pancreatic tissue (Mean±SD; N=3 or 5; two-way anova with multiple comparisons). (e) mRNA levels of *Sox9* assessed by qRT-PCR in saline/caerulein treated xCT WT/KO mouse pancreatic tissue (Mean±SD; N=3 or 5; two-way anova with multiple comparisons). (f) mRNA levels of *Amy2* assessed by qRT-PCR in WT and KO mouse exocrine cell cultures (Mean±SD; N=6; two-way anova with multiple comparisons). (g) mRNA levels of *Krt19* assessed by qRT-PCR in WT and KO mouse exocrine cell cultures (Mean±SD; N=6; two-way anova with multiple comparisons).


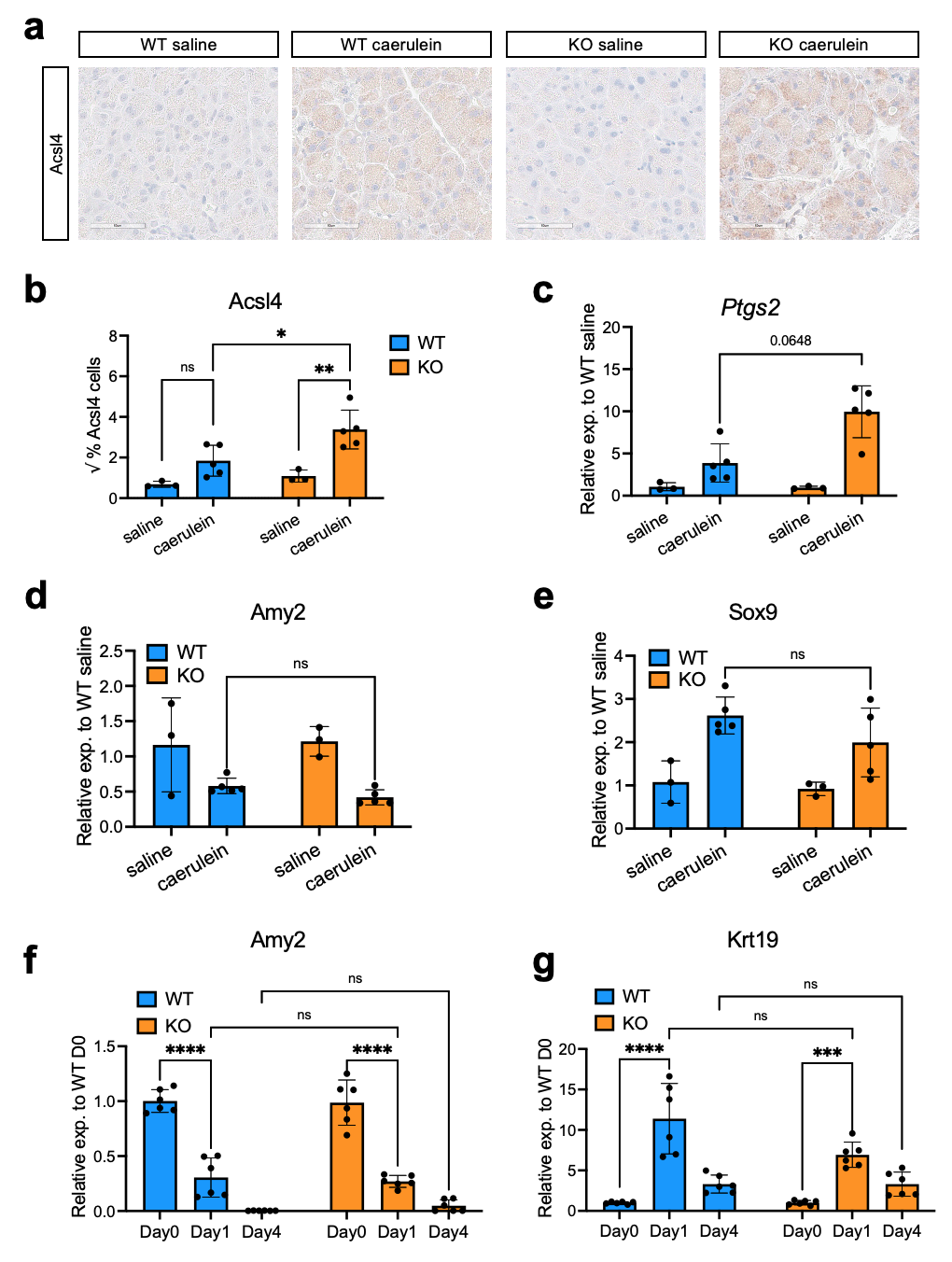


**List of primers**

| mSlc7a11 fwd | AACTGCTCGTAATACGCCCT |
| --- | --- |
| mSlc7a11 rev | AAATCTGGATCCGGGCACTC |
| hSLC7A11 fwd | ATGCAGTGGCAGTGACCTTT |
| hSLC7A11 rev | GGCAACAAAGATCGGAACTG |
| mHprt fwd | GGCCAGACTTTGTTGGATTTG |
| mHprt rev | TGCGCTCATCTTAGGCTTTGT |
| hHPRT fwd | GGCTCCGTTATGGCGACCC |
| hHPRT rev | TGTGATGGCCTCCCATCTCCTT |
| mBhlha15 fwd | CGCTATTTTTCCCCTAGTTC |
| mBhlha15 rev | GGACATTTGTGTTACAGAGG |
| mAmy2 fwd | TGGCGTCAAATCAGGAACATG |
| mAmy2 rev | AAAGTGGCTGACAAAGCCCAG |
| mKrt19 fwd | CCTCCCGCGATTACAACCACT |
| mKrt19 rev | GGCGAGCATTGTCAATCTGT |
| mSox9 fwd | CTAAACTGGTCGAGCGATGG |
| mSox9 rev | TGGTAGGATCATACTCGGAATAG |
| mPtgs2 fwd | GCGACATACTCAAGCAGGAGCA |
| mPtgs2 rev | AGTGGTAACCGCTCAGGTGTTG |

**List of siRNAs**

| xCT siRNA1 | 5’-rArGrC rArArU rUrCrU rGrArU rArArU rUrArU-3’  5’-rArGrG rGrArC rUrArU rArArU rUrArU rCrArG-3’ |
| --- | --- |
| xCT siRNA2 | 5’-rGrGrA rCrUrG rArUrU rUrArU rCrUrU rCrGrA-3’  5’-rGrUrU rUrGrU rArUrC rGrArA rGrArU rArArA-3’ |
| xCT siRNA3 | 5’-rGrArU rUrGrA rArCrA rUrUrU rCrUrC rUrUrA-3’  5’-rUrGrC rUrUrA rCrUrA rArGrA rGrArA rArUrG-3’ |

**SUPPLEMENTARY MATERIALS AND METHODS**

**Cell Line Culture**

The mouse partially differentiated acinar cell line 266-6 (CRL-2151, ATCC) was cultured in DMEM + GlutaMax medium (Gibco), supplemented with 10% heat-inactivated fetal bovine serum (10270106, Thermo Fisher Scientific) and 1% penicillin-streptomycin (15-140-122, Gibco). The cells were kept in a humidified incubator at 37 °C and 5% CO_2_ in the air. The cells were tested for mycoplasma contamination every 6 months and all results were negative. Sulfasalazine (S0883, Sigma-Aldrich) and erastin (S7242, Selleckchem) were used as system x_C_^-^ inhibitors. Stock solutions of these inhibitors were prepared with dimethyl sulfoxide (DMSO) and the final concentration of DMSO in all the assays was less than 0.1%. 2-Mercaptoethanol (31350010, Gibco), Z-VAD-FMK (S7023, Selleckchem) and ferrostatin-1 (Fer-1) (SML0583, Sigma-Aldrich) were used as rescuing reagents and were used at a final concentration of 50μM, 50μM and 1μM respectively. The cell morphology and growth were monitored by IncuCyte Zoom System (Essen Bioscience).

**ROS measurement**

Overall ROS was measured by Fluorometric Intracellular Ros Kit (MAK143, Sigma-Aldrich) according to the manufacturer’s instructions. Cells were incubated at 37 °C for 1 hour after adding ROS master reaction mix. The fluorescence intensity was measured with following settings: λex= 490 nm and λem= 525 nm. The ROS fluorescence value was normalized to cell viability by Cell titer Glo.

**Seahorse Analysis**

Dedifferentiating WT and KO acinar cells at day 1 were subjected to the XF Real Time ATP Rate assay (103592-100, Agilent Technologies) for assessment of mitochondrial and glycolytic ATP production using the XFe 96-well plate Seahorse analyzer, according to manufacturer’s instructions. The Seahorse XFe analyzer measures oxygen consumption rate (OCR) and extracellular acidification rate (ECAR) of live cells in real-time. By adding inhibitors of the electron transport chain (1.5μM oligomycin and 0.5μM rotenone/antimycin A automatically injected onto the cells after 18 and 36 min, respectively), a distinction can be made between mitochondrial and glycolytic ATP production. Briefly, on the day of the assay cells were washed with XF RPMI (Agilent), supplemented with 10mM glucose, 1mM pyruvate and 2mM glutamine. The acinar cell suspension (50μL/well) was then transferred to a poly-l-lysine coated (1mg/ml, Sigma) XFe 96-well plate. The plate was centrifuged (200g, 1min) and transferred to a non-CO_2_ incubator (37°C, 30min). XFe culture medium was added to achieve a total volume of 180 uL, followed by another incubation step of 30 min (non-CO_2_ incubator) before transfer into the XFe analyzer. After the experiment, cells were subjected to the Cell titer Glo assay for normalization.

**siRNA Transfection**

Three different siRNA duplexes targeting *Slc7a11* (xCT) were purchased from Integrated DNA Technologies. Transfection complexes were obtained by mixing siRNA and Lipofectamine RNAiMAX (13778-030, Invitrogen) in Opti-MEM medium (Gibco) according to the manufacturer’s protocol. As for negative control (siCtrl), a scramble siRNA duplex was used. Freshly passaged 266-6 cells were mixed with transfection complexes and seeded into 6-well plates or 96-well plates. The final concentration of siRNA was 10nM. 2-Mercaptoethanol (31350010, Thermo Fisher Scientific) was supplemented to the culture medium for the first 24 hours to improve the viability of the cells. The medium was then replaced by fresh medium without 2-Mercaptoethanol for another 24-hour culture. Cells were harvested or examined 48 hours after transfection.

**Immunohistochemical Staining**

Pancreas tissue and cell pellets were fixed for 24 hours at room temperature in 4% PFA (4078.9010, VWR). Next, samples were washed twice with PBS, dehydrated, and embedded in paraffin. Cell pellets were prepared in agarose before entering paraffin. Sections of 4µm thickness were cut. The primary antibodies were incubated overnight at 4°C. The next day, slides were incubated for 30 minutes with biotinylated secondary antibodies, followed by Streptavidin-biotin-HRP complex incubation, DAB incubation and hematoxylin counterstaining. The following primary antibodies were used: anti-cleaved caspase 3 (9661, Cell Signaling Technology, 1/200), anti-4-HNE (ab46545, Abcam, 1/500), anti-Tfrc (ab214039, Abcam, 1/500), anti-FACL4 (ab155282, Abcam, 1/1000), anti-Amylase (sc-46657, Santa Cruz Biotechnology, 1/200), anti-CK19 (Troma III, Developmental Studies Hybridoma Bank, 1/100) and F4/80 Monoclonal Antibody (14-4801-85, eBioscience, 1/50). Trichrome Masson’s staining was performed automated at the Pathology Department of UZ Brussel, Brussels, Belgium. The slides were scanned by Aperio GT450 (Leica) or Axioscan Z1 (ZEISS).

**Image quantification analysis**

Quantifications of immunohistochemical staining and pancreatic acinar cluster size measurements were performed using HALO® image analysis platform software (Indica Labs). For IHC staining, around 500 cells on average per mouse per condition were analyzed for cell pellet sections; and around 1.7 mm^2^ tissue area on average per mouse was analyzed for pancreatic tissue sections. For cluster size measurement, at least 150 clusters per mouse per condition were identified and analyzed. The mean value and standard error of the mean were used for these comparisons.

**Quantitative Reverse Transcription Polymerase Chain Reaction (qRT-PCR)**

Total RNA was isolated from cells using NucleoSpin RNA isolation kit (Macherey-Nagel) or RNeasy Micro kit (Qiagen). RNA concentration was measured by NanoDrop2000 (Thermo Fisher Scientific). cDNA was prepared using the qScript cDNA synthesis kit (QuantaBio). qPCR was performed using PerfeCTa SYBR Green FastMixes (QuantaBio) on a QuantStudio 12K Flex (Thermo Fisher Scientific). The analysis was done using the ∆∆Ct method using *HPRT* as the housekeeping gene. Genes with Ct values above 35 were considered not detected.

**Western Blotting**

Cells were lysed with RIPA buffer (150mM sodium chloride, 1.0% Triton X-100, 0.5% sodium deoxycholate, 0.1% SDS and 50mM Tris pH 8.0), supplemented with protease inhibitor (P8340, Sigma-Aldrich, 1/100 dilution) and phosphatase inhibitor (P2850, Sigma-Aldrich, 1/100 dilution). Protein concentrations were measured by Bradford protein assay kit (Bio-Rad). Equal amounts of proteins were loaded and separated by SDS-PAGE using 10-15% resolving acrylamide gels. Proteins were transferred onto the nitrocellulose membrane. After blocking the membrane with 5% non-fat milk, primary antibodies diluted in 5% bovine serum albumin (BSA) containing Tris-buffered saline Tween-20 (TBS-T) were incubated at 4 °C. The primary antibodies used were anti-xCT/*SLC7A11* (12691S, Cell Signaling Technology, 1/1000), anti-xCT/*Slc7a11* (98051S, Cell Signaling Technology, 1/1000), anti-GPX4 (52455S, Cell Signaling Technology, 1/1000), anti-actin (A5441, Sigma-Aldrich, 1/5000), anti-tubulin (T9026, Sigma-Aldrich, 1/500). The appropriate secondary infrared-conjugated antibodies (LI-COR) were incubated for one hour at room temperature in the dark. Detection was performed using the LI-COR Biosciences Odyssey® Fc Imaging System and analyzed with the Image Studio™ software (LI-COR).

**RNA Sequencing and Data Analysis**

Single-end RNAseq of dedifferentiated acinar cells from WT and KO mice was performed on a NovaSeq platform at VIB Nucleomics Core with TruSeq library prep. Low quality ends (<Q20) were trimmed using FastX 0.0.14. Reads shorter than 35bp after trimming were removed. Using FastX 0.0.14 and ShortRead 1.44.3, polyA reads (more than 90% of bases equal A), ambiguous reads (containing N), low quality reads (more than 50% of the bases <Q25), and artifacts reads (all but three bases in the read equal one base type) were removed. The preprocessed reads were aligned to the reference genome of Mus musculus Ensembl.GRCm38.82 (GRCm3882). Within- and between-sample normalization was corrected for using full quantile normalization with the EDASeq package from Bioconductor. The number of reads was counted in the alignment that overlapped with gene features using featureCounts 1.5.3 (26). FPKM values were determined by dividing for each sample the normalized counts by the total number of counts (in millions). Then for each gene, the scaled counts were divided by the gene length (in kbp). As such we got the number of Fragments Per Kilobase of gene sequence and per Million fragments of library size. Differential expressions were obtained by statistical comparative analysis with the edgeR 3.28.1 package of Bioconductor. KEGG pathway analysis was performed using the KOBAS gene set enrichment tool (27). Heatmap was plotted by https://www.bioinformatics.com.cn (last accessed on 07 Jan 2023), an online platform for data analysis and visualization.
