# Supplementary figures and images for: Pancreatic acinar cell fate relies on system xC- to prevent ferroptosis during stress
Source: Cell Death Dis. 2023 Aug 21;14(8):536. doi: 10.1038/s41419-023-06063-w (PMC10442358; doi:10.1038/s41419-023-06063-w)

**Fig.1f**

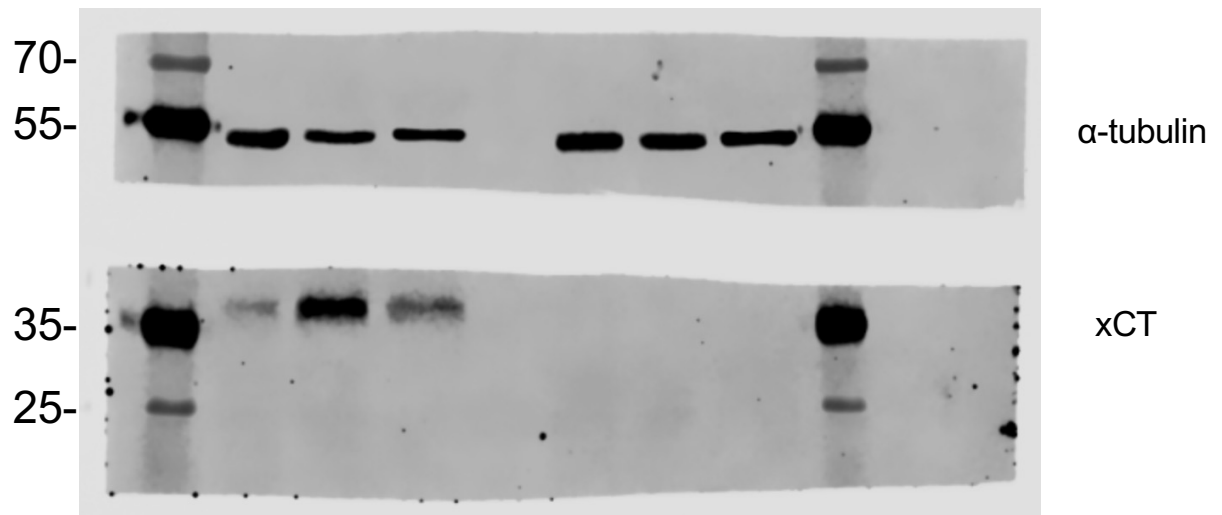

**Fig.1g**

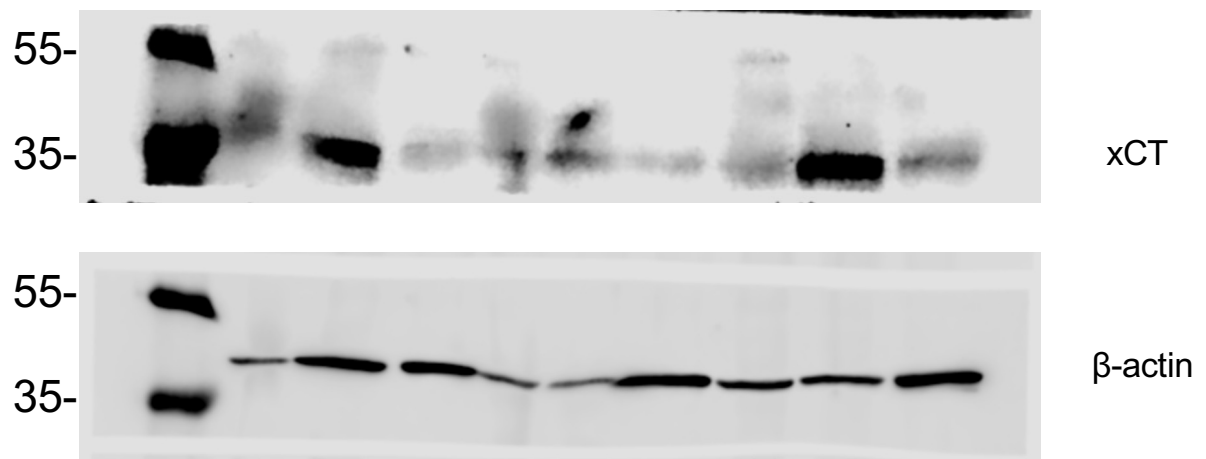

**Fig.3c, h**

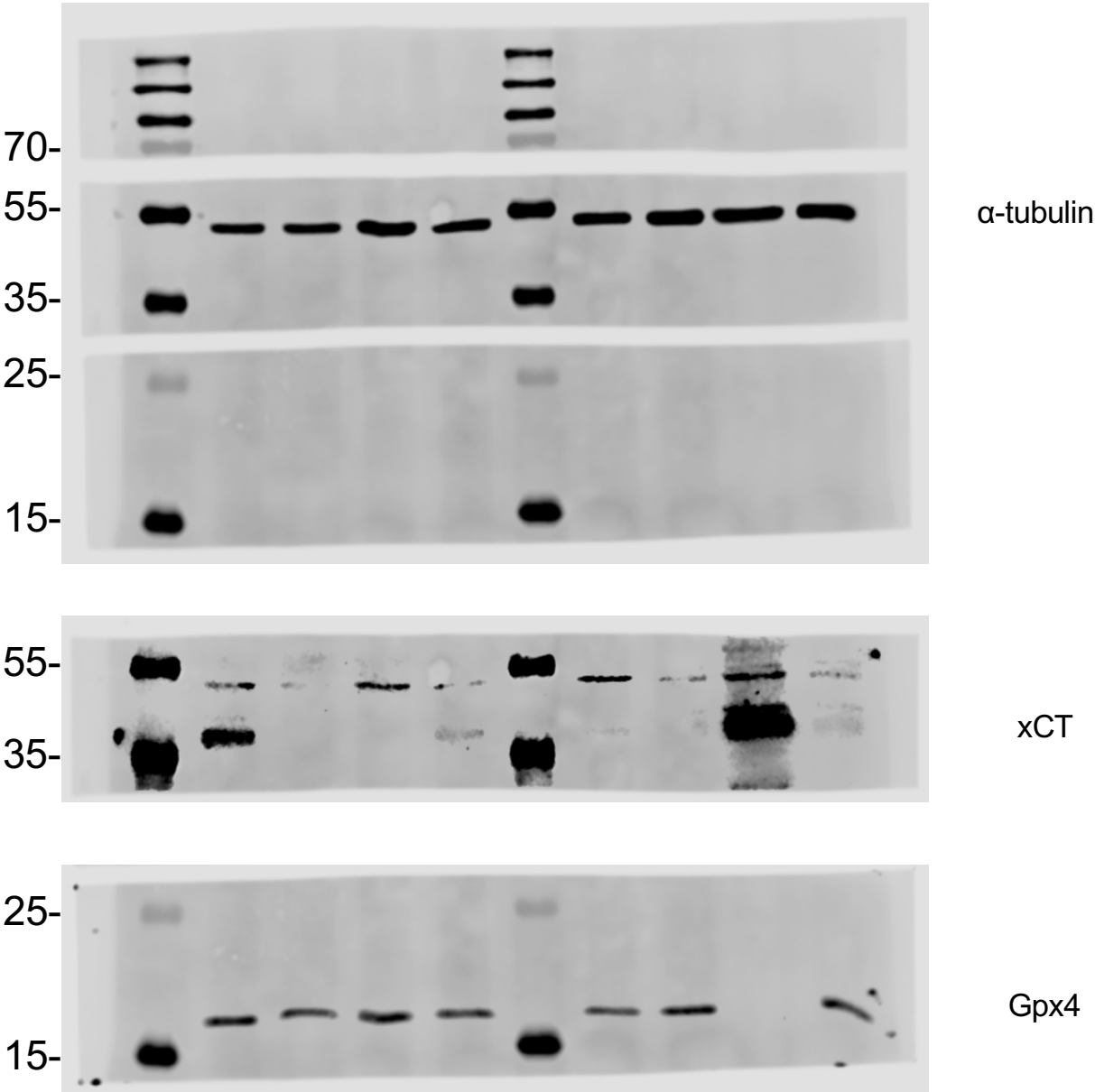

**Fig.4e**

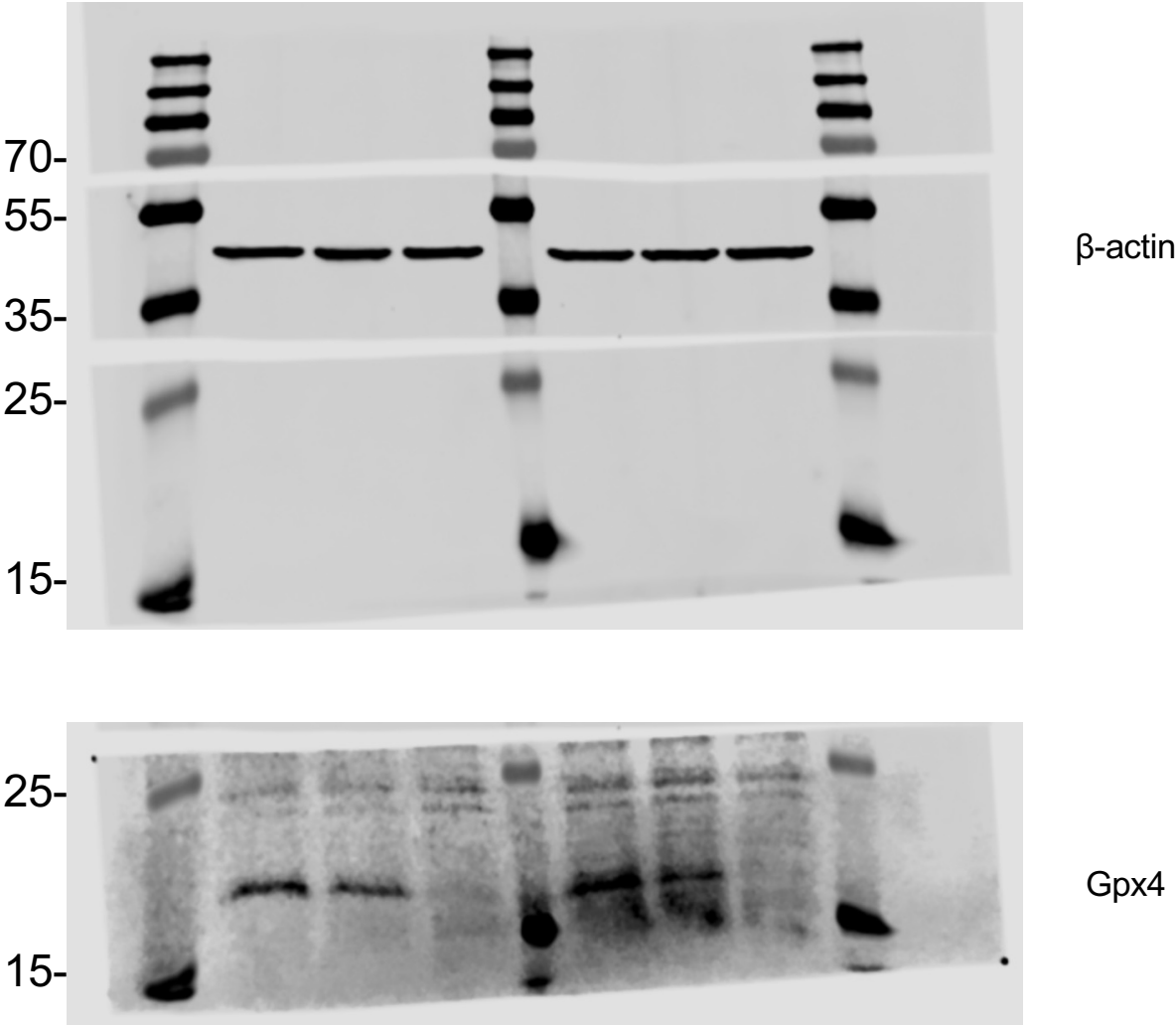

Supplement: Supplementary file 3 — Original Data File [file 41419_2023_6063_MOESM3_ESM.pdf]
